# Cellular ^1^H MR Relaxation Times in Healthy and Cancer Three-Dimensional (3D) Breast Cell Culture

**DOI:** 10.3390/ijms24054735

**Published:** 2023-03-01

**Authors:** Zuzanna Bober, Rafał Podgórski, David Aebisher, Grzegorz Cieślar, Aleksandra Kawczyk-Krupka, Dorota Bartusik-Aebisher

**Affiliations:** 1 Department of Photomedicine and Physical Chemistry, Medical College, Rzeszów University, 35-310 Rzeszów, Poland; 2 Department of Biochemistry and General Chemistry, Medical College, Rzeszów University, 35-310 Rzeszów, Poland; 3 Department of Internal Medicine, Angiology and Physical Medicine, Center for Laser Diagnostics and Therapy, Faculty of Medical Sciences in Zabrze, Medical University of Silesia, 40-055 Katowice, Poland

**Keywords:** cellular MRI, breast cancer, viability, 1.5 Tesla

## Abstract

Noninvasive measurements of ^1^H Magnetic Resonance Imaging (MR) relaxation times in a three-dimensional (3D) cell culture construct are presented. Trastuzumab was used as a pharmacological component delivered to the cells in vitro. The purpose of this study was to evaluate the Trastuzumab delivery by relaxation times in 3D cell cultures. The bioreactor has been designed and used for 3D cell cultures. Four bioreactors were prepared, two with normal cells and two with breast cancer cells. The relaxation times of HTB-125 and CRL 2314 cell cultures were determined. An immunohistochemistry (IHC) test was performed before MRI measurements to confirm the amount of HER2 protein in the CRL-2314 cancer cells. The results showed that the relaxation time of CRL2314 cells is lower than normal HTB-125 cells in both cases, before and after treatment. An analysis of the results showed that 3D culture studies have potential in evaluating treatment efficacy using relaxation times measurements with a field of 1.5 Tesla. The use ^1^H MRI relaxation times allows for the visualization of cell viability in response to treatment.

## 1. Introduction

**Figure 1:**
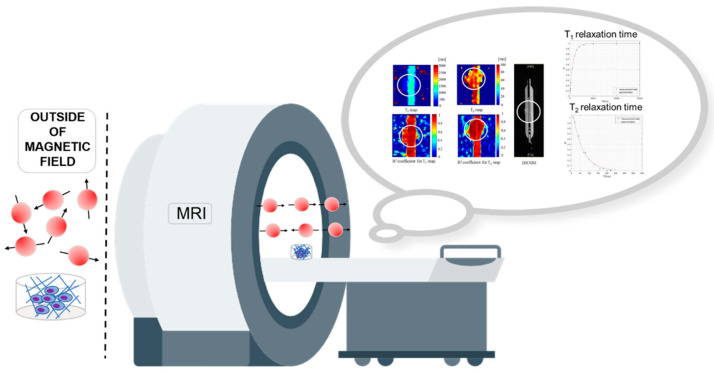
The MRI setup of 3D cell cultures in the bioreactor.

Three-dimensional (3D) cell cultures were used to study the drug delivery. Three-dimensional cell cultures, compared to two dimensional (2D), better mimic the conditions of cancer cells in living organisms. The 3D cell culture reflects physicochemical gradients in the tumor, including extracellular acidity, and increased lactate and limited glucose and oxygen availability to the inner core [1]. The application of flow microsystems in the breeding allows the geometry of microstructures to be designed in a manner to reflect in vivo conditions. The use of 3D cell cultures together with magnetic resonance imaging (MRI) makes it possible to assess cell viability and death in the context of the response to the anticancer drugs used. In addition, the use of cell cultures in research allows for reproducible and repeatable results while providing a high correlation with in vivo conditions. In addition, they allow an assessment of cancer cell biology. Biopharmaceutics studies provide information on the transport of a therapeutic substance through the cell monolayer, or the uptake of this substance by cells in a 3D culture. The preliminary studies on pharmaceutical substances show that the application of MRI for in vitro experimental studies yields satisfactory differences in signal intensity, so it turns out to be important to implement in vitro studies and then try to transfer them to in vivo studies. With the implementation of the method of determining spin–lattice (T_1_) and spin–spin (T_2_) relaxation times, it is possible to assess the condition of cells and check the effectiveness of drug therapy. Using an MRI scanner with a 1.5 Tesla (T) field, we performed a series of measurements, allowing us to determine T_1_ and T_2_ relaxation times in cultures of CRL-2314 breast cancer cells and normal HTB-125 cells. In this study, monolayer cultures of breast cancer cell line CRL-2314 and breast cell line HTB-125 were prepared, as well as 3D cell cultures in a bioreactor for the CRL-2314 and HTB-125 lines. In particular, we wanted to determine whether it is possible to use clinical studies to assess viability during applied therapy based on changes in relaxation times. Analyzing the results obtained, we observed changes in the values of the relaxation times, which may indicate the induction of histone acetylation. MRI provides quality images at a high level, which makes it possible to attempt imaging of small structures, including cell cultures (Figure 1). When using a clinical MR scanner for scientific research, care should be taken to optimize the protocols by selecting appropriate scanning parameters and developing and using a new coil constructed for imaging small objects. In this paper, we will present the results obtained with a new solenoid coil of suitable shape and dimensions. The coil design made it possible to obtain images of optimal quality. MRI is based on the interaction of a strong magnetic field and electromagnetic waves of a certain frequency on hydrogen nuclei. It allows the assessment of the physical properties of the sample, relative to its neighboring areas. In in vitro research, it is used to control the release of drug dosage forms, hydration and diffusion. MR distinguishes between two types of T_1_ and T_2_ relaxation. T_1_ (longitudinal, spin–lattice) relaxation is the time required for the longitudinal magnetization to return to the *Z*-axis at 63% of the original state. The T_1_ relaxation time depends on the temperature, and the speed of the process depends on the force with which the protons interact with the environment. The faster the interaction, the more macromolecules are in the tissue under study. The longest T_1_ relaxation time will be achieved by the sample with the highest water content and the lowest macromolecule content. In contrast, the presence of paramagnetic nuclei in the sample results in a shorter T_1_ time. This phenomenon is used, for example, in the design of gadolinium-based contrast agents. We define the T_2_ relaxation time as the time that must elapse for the transverse magnetization of Mxy to reach 37% of the initial value, and for the spins to lose coherence among themselves. The disappearance of transverse magnetization (T_2_ decay) occurs due to coherence between the spins, hence, this is the spin–spin relaxation time. The implementation of 3D cell culture studies and the popular magnetic resonance diagnostic method continues to attract great interest.

The research presented here was developed to study the influence of Trastuzumab and a Trastuzumab derivative on cells with Her-2 expression close to normal cells. We used quantitative MRI measurements to show the differences in cellular relaxation time in HTB-125 and CRL 2314 cell cultures. We were interested to show the capacity of Trastuzumab to be used in a combined treatment for cells with low Her2 expression.

### 1.1. Experimental Part

All the compounds for the cell cultures were supplied by Fisher Scientific (Oakland, CA, USA). Collagen bovine-type lyophilized fibrous powder from tendon (Advanced BioMatrix, Medley, FL, USA), penicillin–streptomycin–neomycin solution stabilized, fetal bovine serum (FBS), and epidermal growth factor (EGF) were from Sigma-Aldrich, (Sigma-Aldrich, St. Louis, MO, USA). The growth media for the cells were prepared under sterile conditions in a laminar air flow chamber manufactured by Alpina (Konin, Poland). The breast cancer cell line CRL-2314 and healthy cell line HTB-125 (American Type Culture Collection, VA, USA) were used in the experiment. The cultures at the initial stage were conducted in an incubator monolayer in culture bottles until a cell count of 0.5 × 10^7^ cells/mL was reached, and then the cells were seeded in the bioreactors. Four bioreactors were prepared for this research study. The studies used loading doses of the drug, commonly used in therapies in clinical practice, appropriately converted to the volume of the samples. Therefore, a Trastuzumab (Herceptin) solution (20 µg of Trastuzumab per 1 mL of solvent) was added to the cell culture [2]. The Trastuzumab was purchased from Genentech, Inc. (San Francisco, CA, USA) and delivered from the company Roche Polska (Warsaw, Poland). Dendrimer (G5-PAMAM) triethylamine (TEA), heptafluorobutyric acid anhydride (HFAA), sulfosuccinimidyl 6-[3′-(2-pyridyldithio)-propionamido]hexanoate (Sulfo-LC-SPDP), dithiothreitol (DTT), cyclohexane-1-carboxylate (Sulfo-SMCC), methanol, and Sulfo-SIAB were purchased from Sigma-Aldrich, (Sigma-Aldrich, USA).

**Figure 2:**
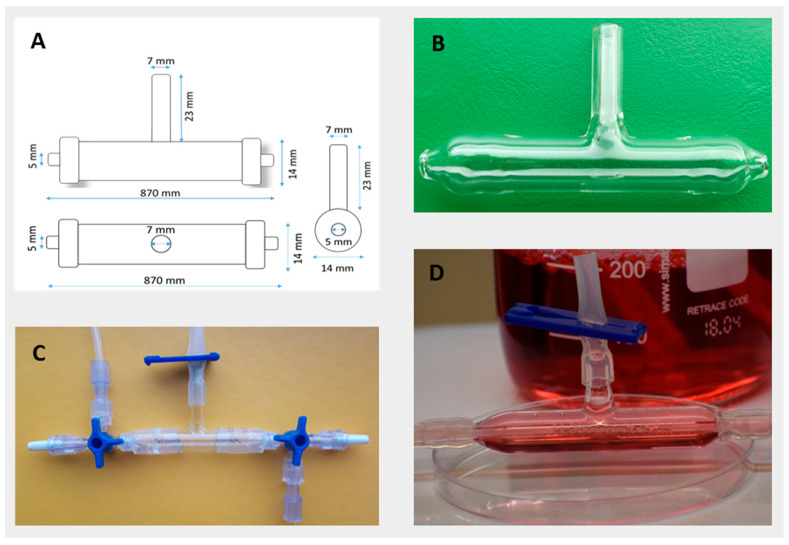
Plans for the bioreactor (**A**); photo of the empty bioreactor (**B**); bioreactor with tubing connection (**C**); and bioreactor filled with culture medium (**D**).

Custom-made glass bioreactors (Figure 2), according to our own design, were used to obtain 3D cell cultures. “Fiber” was made from a blood collection capillary with a diameter of 2.30 mm from Sarstedt AG & Co., Nümbrecht, Germany, in which holes were made so that there was a possibility of accessing the culture medium. The capillary was then gas-sterilized and placed in a sterile bioreactor. The intracapillary space (IC) is the interior of the capillary, while the extracapillary space (EC) is the space where the cells inoculate on the fibers in the bioreactor and expand there. The cell culture medium was in both the IC and EC spaces and is intended to supply oxygen and nutrients to the cells. At the top of the bioreactor is a chimney, in which a 0.22 μm filter was placed, so as to prevent access from the outside by unwanted agents.

In the next step, the fiber was coated with a collagen solution. After the cells were introduced to the device, the bioreactor was supplemented with growth media suitable for the given cell cultures. The cells were incubated in an atmosphere of 5% CO_2_ and a temperature of 37 °C. The laboratory peristaltic pump used for the system was from Lead Fluid Technology (Baoding, China). The breast cancer cells were allowed to grow in the bioreactor until their density reached 10^9^ cells/mL.

In the next step, the following compounds were added to the bioreactors with the 3D breast cancer cell line, CRL-2314, and HTB-125 as a control. The Trastuzumab derivatives (2–5) were obtained previously [2]. The potential detection of HER2 protein in the CRL 2314 was first carried out by IHC, according to the guidelines published by Wang et al. [3].

### 1.2. Bioreactor Setup

**Figure 3:**
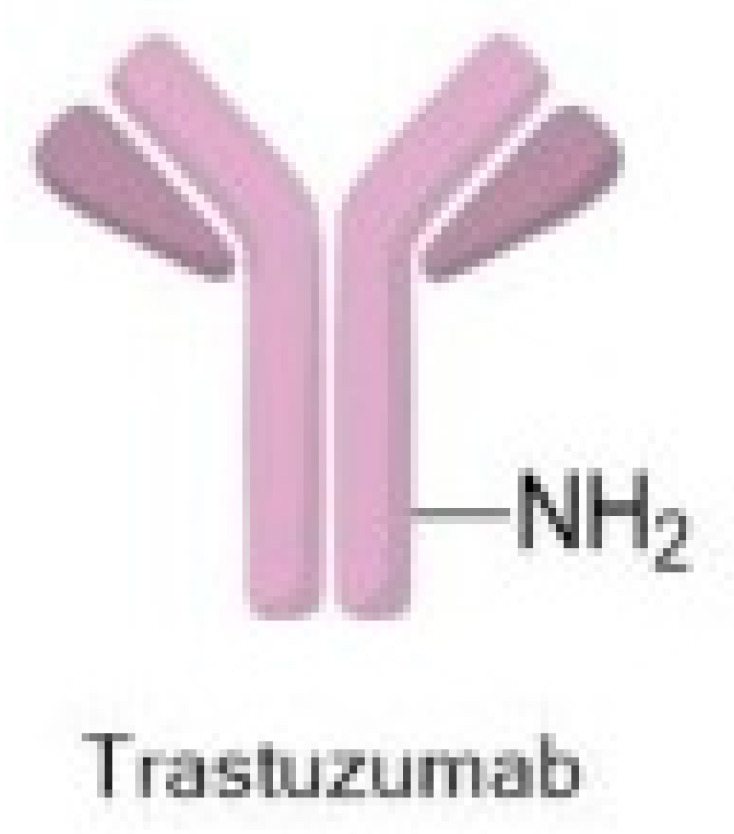
Trastuzumab.

The CRL-2314 cells were introduced to the bioreactor at a density of 10^9^ cells/mL and treated with a 100 µL solution of Trastuzumab (**1**) (Figure 3). We used Trastuzumab of 145,531.86 g·mol^−1^ MW.

**Figure 4:**
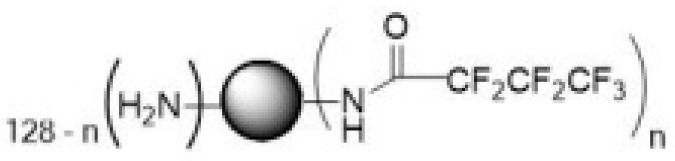
Fluorinated PAMAM G5.

The CRL-2314 cells were seeded in the bioreactor at a density of 10^9^ cells/mL and treated with 100 µL of fluorinated PAMAM G5 solution (**2**) (Figure 4).

**Figure 5:**
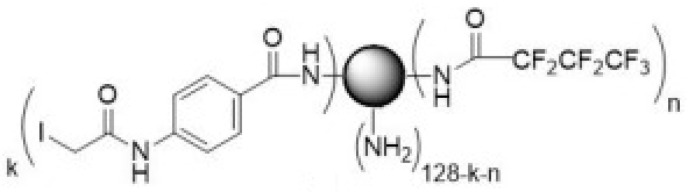
PAMAM G5-Sulfo-SIAB (**3**).

The CRL-2314 cells were seeded in the bioreactor at a density of 10^9^ cells/mL and treated with 100 µL of fluorinated PAMAM G5-Sulfo-SIAB (**3**) (Figure 5).

**Figure 6:**
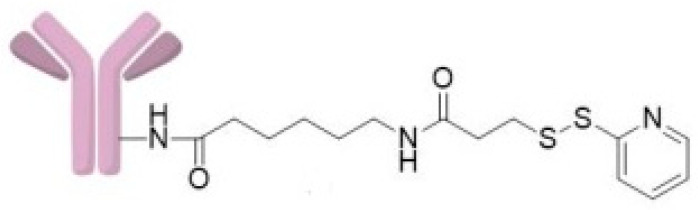
PAMAM G5-Sulfo- LC-SPDP (**4**).

The CRL-2314 cells were treated with 100 µL of Trastuzumab Sulfo-LC-SPDP (**4**) (Figure 6).

**Figure 7:**
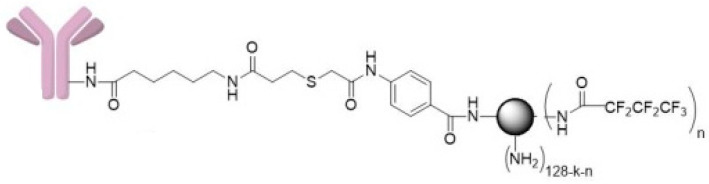
Thioether cross-linked dendrimer conjugate Trastuzumab derivative (**5**).

The CRL-2314 cells were treated with 100 µL of thioether cross-linked dendrimer conjugate Trastuzumab derivative (**5**) (Figure 7).

### 1.3. MTT Assay

A decrease in numbers was noted after the use of Trastuzumab and Trastuzumab derivatives. An MTT assay was used to assess cell viability. This assay is a colorimetric reaction that can easily be measured from cell monolayers that have been plated in 35 mm dishes or multi-well plates. Typically, 10,000 cells suspended in 100 μL of medium are incubated with 10 μL of MTT reagent (Cat # 30-1010 K; ATCC) for approximately 3 h, followed by the addition of a detergent solution to lyse the cells and solubilize the colored crystals. The cell cultures were incubated for 3 h in a culture medium containing 0.5 mg mL MTT reagent. After 2 h, the incubation buffer was removed and the blue MTT–formazan product was extracted with acidified isopropyl alcohol (0.04 N HCl). After 30 min extraction at room temperature, the absorbance of the formazan solution was read spectrophotometrically. After using the therapy, a decrease in vitality was noticed on average at a level of 11%.

### 1.4. Magnetic Resonance Imaging

Longitudinal (T_1_) and transverse (T_2_) relaxation times were measured using a 1.5 T magnetic resonance scanner with a dedicated transmit/receive coil. The prepared specimens were scanned using a fast spin echo (FSE) sequence with axial projection using a small flex coil. On the basis of the obtained DICOM images, an analysis was performed, and ROI measurements were made for a series of images for each sample, based on which T_1_ and T_2_ relaxation times were determined. The technical parameters for the study using the magnetic resonance camera were the same for all the stages of the study. The scanning matrix was 320 × 224, the field of view (FOV) was 6 cm × 6 cm and the section thickness was 1 mm, the spacing was 0.5 mm, and NEX = 2.

For T_1_ time, measurements were performed in 12 steps with a repetition time (TR) ranging from 50–15,000 ms (50, 100, 200, 500, 700, 1000, 1500, 2000, 3000, 5000, 10,000, 15,000 ms) with a constant echo time (TE) of 3 ms. On the basis of the obtained DICOM images, an analysis was performed, and ROI measurements were made for 12 images for each sample, from which the T_1_ relaxation time was determined.

On the other hand, for T_2_ relaxation time, a series of 12 steps was performed keeping the same scanning parameters except for the repetition time, which was fixed at 10,000 ms, the echo time ranging from 11.8–300 ms (11.8, 20, 42, 68, 85, 102, 102, 130, 160, 200, 230, 260, 300 ms), and NEX = 3. Increasing the value of NEX increases the SNR and scan time.

After the test was performed, the prepared samples were incubated for 24 h at 5% CO_2_ and 37 °C. After this time, the entire study was repeated with exactly the same settings and the same scanning parameters.

The results are presented as the mean ± standard deviation (SD), based on independent experiments. The statistical analysis was done by a one-way analysis of variance (ANOVA) using Statistica 10.0 software (StatSoft, Kraków, Poland). A probability level of *p* < 0.05 was considered significant.

## 2. Results

The breast cancer cell line CRL-2314 and the normal cell line HTB-125 were evaluated. The results obtained are presented below (Table 1). In addition, the relaxation times were measured for the prepared Trastuzumab solution, the average value of which was T_1_ = 3334.31 ms and T_2_ = 147.7 ms, respectively. The CRL-2314 cell line showed low-HER2 expression. The low Her2 expression in the CRL-2314 cells was confirmed, based on the IHC test and the MRI experiment. The detection of Her-2 was weak but definitive; staining of the membrane of over 100% of the cytoplasmic circumference was noticed in 30% of the neoplastic cell population.

**Figure 8:**
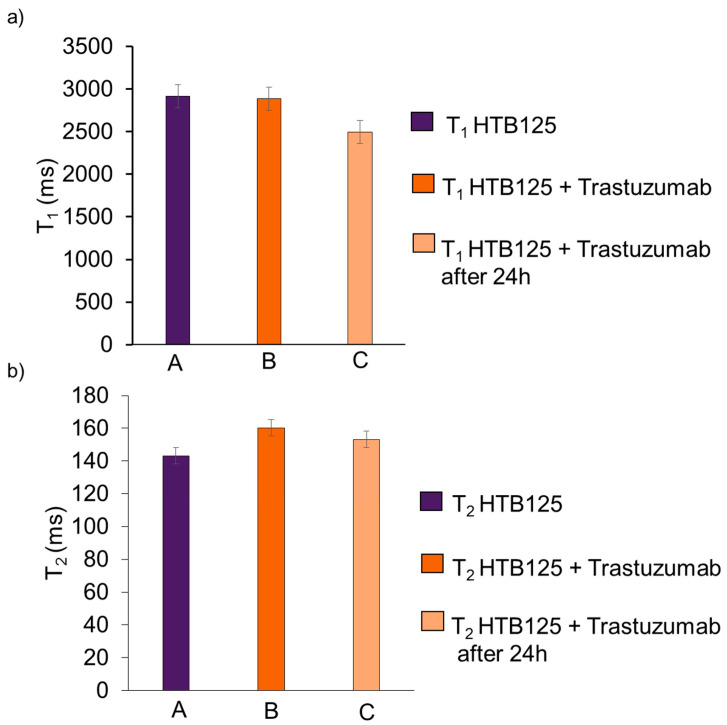
Results of T_1_ (**a**) and T_2_ (**b**) relaxation times for HTB-125 cell culture (A), and the same culture with the addition of Trastuzumab (B), along with the results after 24 h (C).

The graphs below show the results of the relaxation times for the HTB-125 cell culture and the same culture with the addition of Trastuzumab, and the results after 24 h (Figure 8).

**Figure 9:**
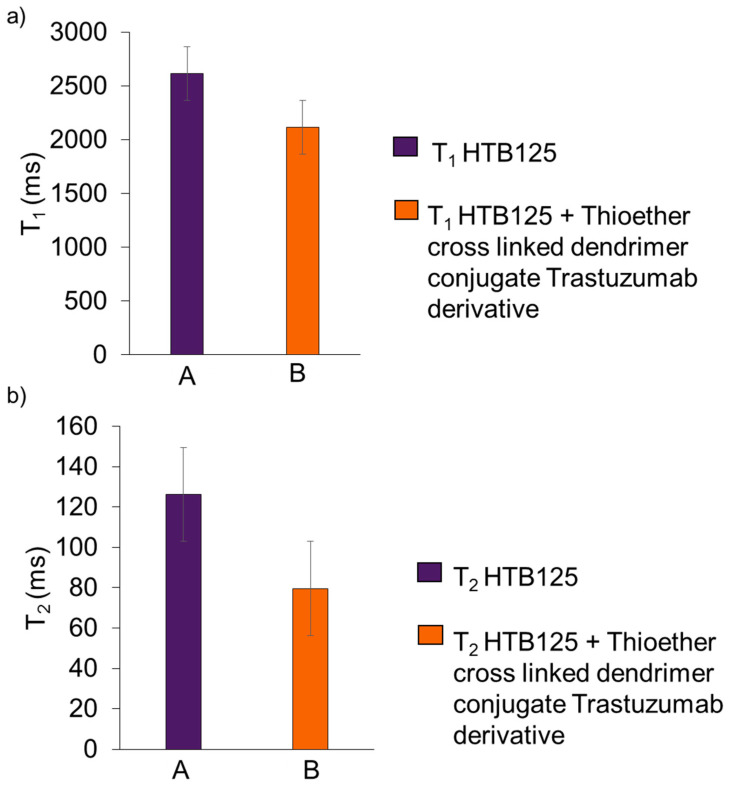
Results of T_1_ (**a**) and T_2_ (**b**) relaxation times for HTB-125 cell culture (A), and the same culture with the addition of Thioether cross linked dendrimer conjugate Trastuzumab derivative (B).

The graph below shows the results of relaxation times for the HTB-125 cell culture, and the same culture with the addition of synthesized drugs Trastuzumab, along with the results after 24 h (Figure 9).

In the next step, the following portions of compounds were added to four prepared bioreactors with CRL-2314 breast cancer cells (Table 2).

The results of T_1_ and T_2_ relaxation times for the bioreactors with the HTB-125 and CRL-2314 cell lines and samples of the same cell lines with the addition of the synthesized trastuzumab–dendrimer–fluorine drug are shown in Table 2.

**Figure 10:**
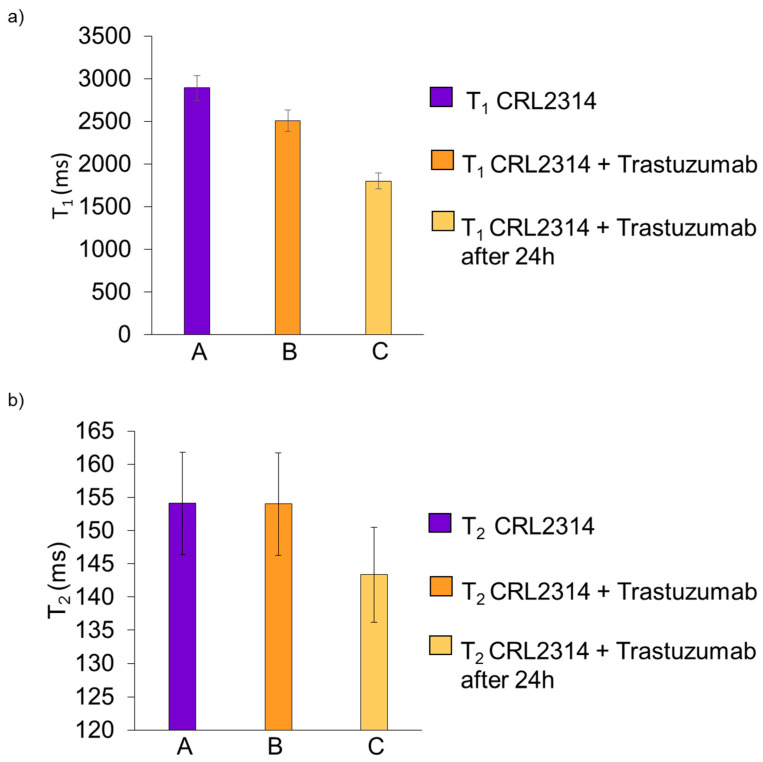
Results of T_1_ (**a**) and T_2_ (**b**) relaxation times for CRL-2314 cell culture in a 3D bioreactor (A), and the same culture with the addition of Trastuzumab (B), along with the results after 24 h (C).

The graph below shows the results of the relaxation times for the CRL-2314 cell culture, and the same culture with the addition of the drug Trastuzumab, and the results after 24 h (Figure 10).

**Figure 11:**
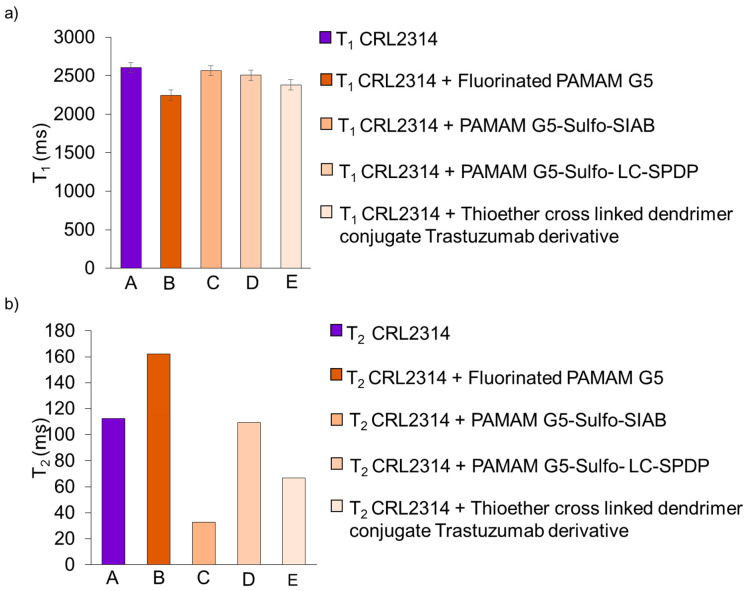
Results of T_1_ (**a**) and T_2_ (**b**) relaxation times for CRL-2314 cell culture in a 3D bioreactor (A), and the same culture with the addition of synthesized drugs 2 (B), 3 PAMAM G5-Sulfo-SIAB (C), 4 PAMAM G5-Sulfo- LC-SPDP (D) and 5 Thioether cross-linked dendrimer conjugate Trastuzumab derivative (E).

The graph below shows the results of relaxation times for the CRL-2314 cell culture and the same culture with the addition of the drug Trastuzumab, along with the results after 24 h (Figure 11).

**Figure 12:**
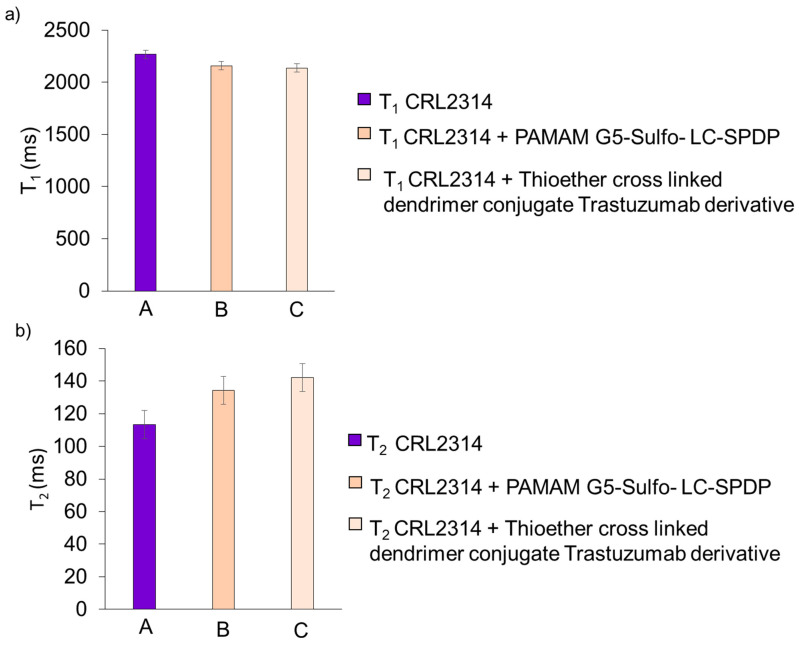
Results of T_1_ (**a**) and T_2_ (**b**) relaxation times for CRL-2314 monolayer cell culture (A), and the same culture with the addition of synthesized drugs 4 PAMAM G5-Sulfo- LC-SPDP (B) and 5 Thioether cross-linked dendrimer conjugate Trastuzumab derivative (C).

The results of the relaxation times for the cultures of the CRL-2314 cells and cells of the same lineage with the addition of drugs 4 and 5 are shown below (Figure 12).

**Figure 13:**
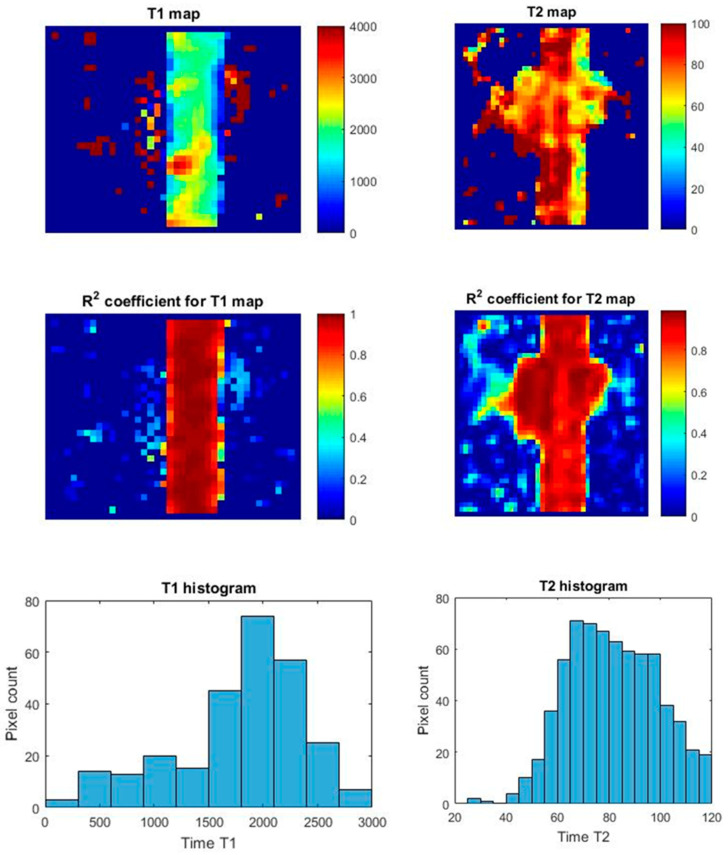
Maps of T_1_ and T_2_ relaxation times for the HTB-125 bioreactor +5 Thioether cross-linked dendrimer conjugate Trastuzumab derivative in the bioreactor, along with R^2^ coefficient and histograms.

**Figure 14:**
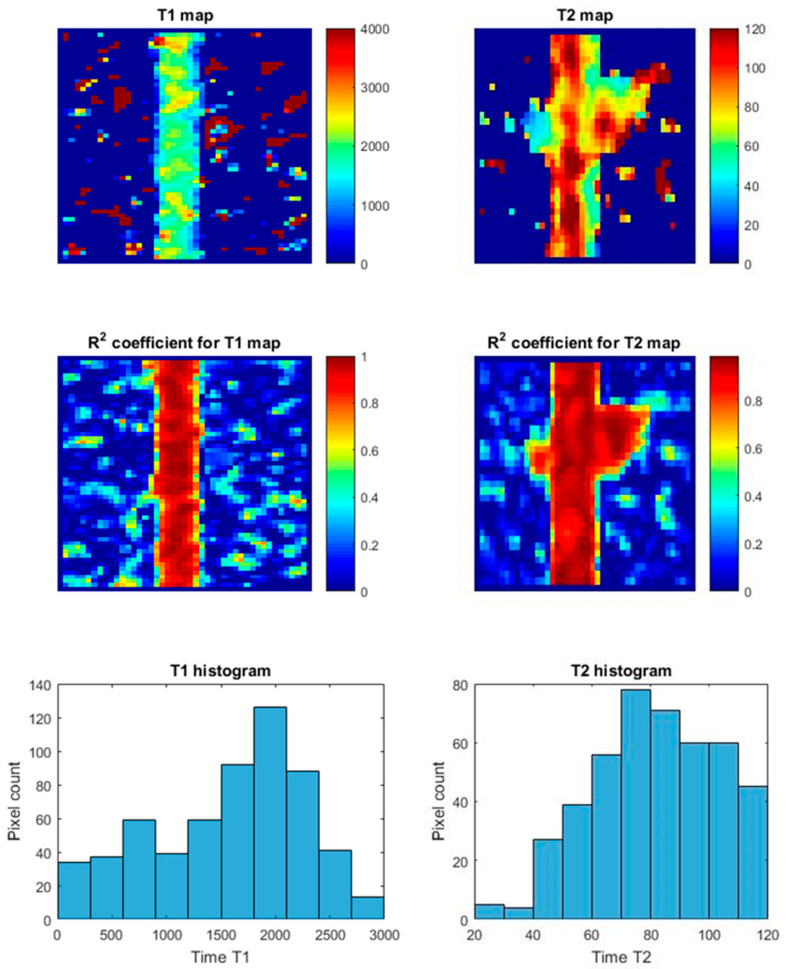
Maps of T_1_ and T_2_ relaxation times for the CRL-2310 bioreactor +5 Thioether cross-linked dendrimer conjugate Trastuzumab derivative in the bioreactor along with R^2^ coefficient and histograms.

The implementation of measurements using cultures in bioreactors made both qualitative and quantitative measurements possible. Based on the measurements, maps of T_1_ and T_2_ relaxation times were created. Example relaxation time maps for the HTB-125 and CRL-2314 cell lines are shown below in Figure 13 and Figure 14, respectively.

## 3. Discussion

The incidence of breast cancer is increasing every year [4]. Still, the results of the applied therapies are unsatisfactory. Therefore, there is a need to implement studies that allow the evaluation of the therapies used and an assessment of tumor metabolism. HER2+ receptor overexpression is of great interest because it is found in about 20% of patients. Overexpression or amplification of HER2 makes it possible to predict the effectiveness of treatment with Trastuzumab. This is a monoclonal antibody directed against HER2 [5]. The importance of the HER2 receptor in the pathogenesis of breast cancer and the implementation of studies using magnetic resonance imaging (MRI) allows, in a non-invasive way, an assessment of the therapeutic response, and thus allows us to predict the course of the cancer. The diagnostic evaluation of cancers with HER2 overexpression and research in genomics, epigenetics, transcriptomics, and proteomics continues to be of great interest to investigators and of great value. MRI research is a rapidly developing field with strong translational and research potential, enabling cell labeling, among other things. Detecting and tracking cells and therapies makes it possible to study disease processes and monitor the therapies used. Finding molecular changes in metabolic pathways can be helpful in selecting the appropriate treatment regimen, and thus have an impact on therapeutic response. Understanding and learning about the complexities of cancer biology are possible by implementing studies using cell cultures. Breast cancer tumors is a heterogeneous group, both at the clinical and molecular levels. HER receptors (HER1, HER2, HER3, HER4) play an important role in the pathogenesis of breast cancer. Too many copies encoding the ERBB2 gene of the HER2 protein affect its overexpression in the cancer cell [6]. The HER2 transmembrane molecule is formed by a gene encoding located on 17q21.1, and its overexpression of HER2 occurs in about 20% of breast cancer cases. It is characterized by a worse prognosis and significantly affects the aggressiveness of the cancer course and the therapy used. Immunotherapies used in the treatment are usually based on monoclonal antibodies, such as Trastuzumab class IgG1, which has a therapeutic effect against the positive HER2 domain. Currently, the biological drug Trastuzumab is thought to be effective only in patients with cancer with HER2 overexpression [7]. Currently, there is a search for markers that would allow for predicting the development of the tumor, as well as selecting an effective treatment regimen, individually tailored to the patient. The HER2 receptor is a member of a family of four closely related growth factor receptors (EGFR/HER1/erbB-1, HER2/erbB-2, HER3/erbB-3 and HER4/erbB-4). Overexpression of the HER2 receptor is caused by the amplification of the HER2 gene, which is found in epithelial tumors, including breast cancer. It causes rapid disease progression due to excessive cell proliferation. HER2 is treated as a molecular prognostic factor in the understanding of cancer biology, making it complementary to macroscopic and microscopic evaluation. Treatment with the drug Trastuzumab, which targets the HER2 protein, allows the inhibition of proliferation. The antibody binds to the HER2 receptor without stimulating it. The HER2 receptor is not the only factor that determines proliferation, but Trastuzumab inhibits the effects of many stimulated growth factor receptors. Particular attention should be paid to the classification of tumors into tumors that do not overexpress the HER2 receptor. HER2-negative cancer is when HER2 expression is scored 0+ or 1+ or 2+ by IHC without ISH gene amplification. HER2-low breast cancer is gaining increasing research interest [8]. The concept of “HER2-low” in breast cancer has been proposed to refer to breast cancer with a HER2 IHC score of 1+ or 2+/ISH-negative [9]. In 2022, the U.S. Food and Drug Administration (FDA) approved a targeted therapy using T-Dxd to treat patients with unresectable or metastatic HER2-low breast cancer levels [10]. Compared to HER2-0 breast cancer, HER2-low breast cancer is characterized by a higher expression of ERBB2 mRNA, as well as an increased frequency of the PIK3CA mutation and a decreased TP53 mutation [11]. Modi Group et al. conducted a phase 3 analysis in a cutting-edge study involving patients with low-metastatic HER2 breast cancer who had received one or two previous lines of chemotherapy. The patients received trastuzumab derukstecan or chemotherapy. In a group of patients with low metastatic HER2 breast cancer, trastuzumab derukstecan resulted in significantly longer progression-free and overall survival than chemotherapy [12]. Research into developing new HER2-targeted ADCs and identifying their clinical benefit in HER2-low breast cancer may contribute to improving the clinical treatment of breast cancer.

When 2D cell cultures are conducted in a laboratory setting, although they provide a lot of information at low concentration of cells, compared to 3D cultures, and do not reflect the basic characteristics of the tumor. To reproduce the tumor microenvironment, science has developed 3D cultures of tumor cells. This makes it possible to reflect in vivo conditions, where, in addition, there is a complex and dynamic process of communication on the cell–cell line and cell–matrix interactions. The use of a 3D culture makes it possible to create an environment that corresponds to the morphology of the tumor in vivo. An example of 3D culture is a culture in an HFB-type bioreactor, which allows us to obtain a high density of cells that grow in a controlled environment. As a result, we have achieved high reproducibility and the possibility of implementing studies on tera-therapeutic substances, for example. In a 3D culture, we have interactions between neighboring cells and the extracellular matrix (ECM). These interactions affect the biochemical and mechanical signals of the cell’s physiology, whereas with cells cultured in 2D, this interaction is absent. The use of 3D methods allowed us to acquire information about the culture and introduce imaging studies using MRI. We used MRI imaging and prepared CRL-2314 breast cancer cell cultures treated with the synthesized drug to evaluate Trastuzumab–fluorine–dendrimer drug delivery. Trastuzumab is known to be of clinical benefit in the treatment of breast cancer cells overexpressing Her-2. Her-2 receptors provide the opportunity for active and specific drug targeting and monitoring. For more detailed information on the binding of the drug to the Her-2 receptor, in our study, we implemented a treatment for CRL-2314 and control HTB-125 cells. The current study provides valuable information on the use of non-invasive imaging, such as MRI, to evaluate breast cancer cell cultures induced with Trastuzumab or its modifications under laboratory conditions [13,14,15]. Fluorinated PAMAM G5 dendrimer, fluorinated PAMAM G5-Sulfo-SIAB, Trastuzumab Sulfo-LC-SPDP, and Trastuzumab derivative were used for the measurements. The MRI showed changes in relaxation time values between the T_1_ relaxation time values for the cultured CRL-2314 breast cancer cells and HTB-125 breast cells compared to the same cells after the Trastuzumab drug delivery. For the T_2_ relaxation times, the differences were on the order of 11% for the HTB-125. Tracking drugs using MRI continues to be a challenge. In recent years, the use of drugs in MR imaging has been extensively evaluated in terms of therapeutic effect and drug delivery within the lesion. In addition, the solubility and absorption of synthesized fluorine-containing molecules have proven to be a problem. Modification of the drug Trastuzumab may help improve the efficacy of therapy by improving the bio-distribution and absorption of the drug. However, it should be noted that the process of modifying, synthesizing and combining with Trastuzumab is complicated and very lengthy. However, it should be noted that MR imaging is a non-invasive test, which enables the acquisition of high-resolution images. ^1^H MRI measurements were taken to determine cell cultures (2D) and their morphology (3D). By implementing measurements using cultures in bioreactors, both qualitative and quantitative measurements were possible. Studies of pharmaceuticals in cell cultures are extremely valuable because they indicate the dependence and susceptibility of cancer cells. Implementing 3D cell culture research helps to achieve conditions imitating in vivo conditions, which can greatly improve the development of an effective drug delivery system. The preliminary studies on pharmaceutical substances show the possible application of MRI for cell culture studies. Our results show that it is possible to image cell cultures using a 1.5 Tesla field, and the implementation of non-invasive imaging studies using MRI with Trastuzumab could have an impact on the development of drug delivery systems and influence the improvement of drug efficacy by finding the optimal drug modification. By implementing measurements using cultures in bioreactors, both qualitative and quantitative measurements were possible. Based on the measurements, maps of the T_1_ and T_2_ relaxation times were created. In addition, the implementation of studies using ^19^F MRI makes it possible to obtain a satisfactory signal in the ^19^F MRI in the studied sample with a very high density of ^19^F nuclei, enabling imaging without the tissue background.

### 3.1. Cellular Magnetic Resonance Imaging (MRI)

Magnetic resonance imaging is one of the rapidly developing analytical methods. It allows the acquisition of data without the use of harmful factors, and it enables the imaging of objects and organisms on a micro- and macro-scale. Although MRI is a common imaging method used in medicine, its potential and development in research have not yet been fully exploited. A constantly developing field of MRI is cellular MRI [16], which has strong translational and research potential. The detection and tracking of cells in in vivo tests allows the assessment of cellular processes triggered by the pathogenic process. In addition, it allows the monitoring and tracking of applied therapies. Unfortunately, the use of cellular MRI has many limitations, especially with the use of a clinical MRI scanner. The development of cell labeling methods and their various applications continue to be of great interest among researchers. The continuous evolution of medical imaging, successive versions of software and generations of equipment allow for the continuous development of advanced diagnostics. In addition to morphological and functional imaging, there are possibilities for imaging with the use of specific contrast agents, which allows for cellular imaging. Research has seen an increase in the use of MRI techniques for imaging at the molecular and cellular levels. Moreover, cellular MRI brings us closer to the simultaneous monitoring of the applied therapies. In contrast, single-particle MRI imaging is extremely important in cellular imaging. The improvement of the MRI signal through the use of contrast agents, which significantly improves relaxation, is significant in this type of research. Contrast agents have found their use in the MRI method because of their properties in significantly improving the quality of images. Due to the cumulative contrast medium, depending on the sequence performed, we can obtain two types of images: hyperintense (T_1_-dependent images) and hypointense (T_2_-dependent images). It is extremely important in obtaining better contrast between different tissues, healthy and pathologically changed, and it is also extremely important in cellular imaging. Improvement of the MRI signal can be achieved with paramagnetic or superparamagnetic particles affecting the image contrast. Examples of positive contrast agents are agents based on gadolinium (Gd) [17,18] or manganese (Mn) [19,20], while negative ones are based on iron oxide [21]. In MRI imaging, SPIO molecules are very often used, resulting in a reduction in T_1_, T_2_ and T_2_*. An example of a contrast agent used in cellular imaging is dextran-coated nanometer ultra-light iron oxide particles (USPIO) [22]. These are superparamagnetic molecules that improve the MRI signal. Their usefulness has been presented in numerous publications. They are used, among other things, for the detection of receptors, as well as for monitoring cell migration. The implementation of cell therapies and cellular MRI makes it possible to follow the fate of therapeutic cells and check the therapeutic benefit. In numerous studies on animal models, they allowed for simultaneous imaging and optimization of applied therapies [23]. MRI examinations with the supply of contrast agents based on heavy metals (SPIO and Gd) are becoming more and more popular, together with the imaging of magnetic particles (MPIO) [24,25] and fluorine (^19^F MRI) [26,27]. Improving the sensitivity, which allows the detection of a single cell, is possible with specific scan parameters while saving the cells. Studies have shown that labeling cancer or stem cells does not cause cell toxicity [28]. The detection of SPIO molecules is limited by their low specificity, while in vivo imaging faces many limitations. Despite this, SPIO is used in scientific research to track many types of cells, including cancer cells [29], stem cells [30,31], including human neural stem cells [32], as well as resistance cells [33]. In their work, Ghanbarei et al. evaluated human mesenchymal stem cells labeled with superparamagnetic zinc-nickel ferrite nanoparticles. Their studies showed no negative impact on NFNP dextrin-coated cells, thus suggesting their potential clinical efficacy [34]. In addition, it has been shown that labeling immune cells with micron-sized iron oxide (MPIO) particles (0.35, 0.90 or 1.63 microns) results in a higher MPIO uptake of macrophages for larger particles. A greater T_2_ relaxation was also demonstrated for MPIO-labeled cells as compared to nanometer-sized USPIO-labeled cells with similar iron content [35].

### 3.2. Cellular MRI in Endocrinology

Magnetic resonance imaging is the examination of choice for the assessment of endocrine diseases. Most often, these diseases are associated with the hypothalamus and pituitary gland. The role of imaging methods in the assessment of pituitary–hypothalamic axis changes is extremely important in pumpkin and is the most frequently chosen method [36] also in pediatric studies [37]. The use of paramagnetic and superparamagnetic micelles, or lipid-based nanoparticles, as an MRI contrast agent enables molecular imaging [38]. The implementation of isolated cells, loaded with a contrast agent by in vitro incubation before systemic administration or direct administration of superparamagnetic iron oxide particles into the circulatory system, allows their use in molecular tests for imaging neurological inflammation after patients after stroke using MRI [39]. The research presented multifunctional FE_3_O_4_@SiO_2_(Gd-DTPA)-RGD NP nanoparticles for in vitro and in vivo MR imaging. The results show that they enable targeted double-contrast MR imaging of T_1_ and T_2_ tumor cells overexpressing α (v) β (3) integrin in vitro and in vivo [40]. In addition, MRI enables the tracking of SPIO-labeled monocytes in in vivo studies. The enhancement of the contrast after injection of free USPIO did not primarily represent signals from peripherally labeled monocytes that migrated towards the inflammatory lesion. The use of SPIO-labeled monocytes provides a better tool for a detailed assessment of the monocyte infiltration time window [41]. In other studies, the use of SPIO for post-epileptic imaging of the inflammatory process in an animal lithium–pilocarpine model was presented [42]. Nanoparticles are used as MRI contrast agents for stem cell tracking, as well as for the potential treatment of cardiovascular disease [43]. The publications also show MRI tracing of neural progenitor cells (NPCs) in an undamaged brain in in vivo animal studies. The cells labeled with superparamagnetic iron oxide particles were imaged during implantation and after the initiated stroke [44].

The first measurements of spin–ratio relaxation times using FLASH for dynamic quantitative T1 relaxation maps were obtained before and after a bolus of gadolinium contrast. This made it possible to calculate the concentration–time curve of the paramagnetic factor in breast tissue [45]. The estimation of T2* relaxation time and correlation of the results with the clinical features showed that tumor groups with higher histological grades showed a longer T2* relaxation time [46]. The study also presents a novel qMR method, synthetic MRI (syMRI), which enables the simultaneous generation of T_1_ and T_2_ maps with a single scan. Quantitative mapping derived from synthetic MRI (syMRI) shows high potential for breast cancer diagnosis. Differences in T_1_ and T_2_ times can distinguish between benign and malignant breast lesions [47]. The T_2_ relaxation times of benign and malignant breast lesions in 67 patients were evaluated. During the study, a sensitivity of 85.7% and a specificity of 58.7% were obtained. The results show a significant difference in T_2_ relaxation times between benign and malignant breast lesions [48]. Implementing the results from T_2_ relaxation time to differentiate benign from malignant lesions may be a new addition to breast cancer MRI diagnosis. Other studies show the use of a 3T MR scanner and T2* assessment. Dynamic contrast-enhanced (DCE) MRI and diffusion-weighted imaging (DWI) parameters are markers for breast cancer detection. Also, T2* relaxation time has been proposed as an imaging biomarker in cancer. However, studies show differences in the relationship between T2* and contrast agent uptake in responders and non-responders. The T2* of breast tumors in the first phase of the study is not associated with DCE and DWI parameters and contributes to the description of the functional heterogeneity of breast tumors [49]. The study shows that the use of T2* relaxation times of single mammary ducts in a mouse model may have applications in identifying early invasive tumors. Developing a test scheme and transferring it to diagnosis among patients has a very high potential for imaging diagnosis of early breast cancer in patients using T2∗ [50]. Recent preliminary studies report native T_1_ mapping of the breast on MRI to distinguish adenomas from benign phyllodes tumors. Benign phyllodes tumors were characterized by longer T_1_ relaxation times compared to fibroadenoma tumors, demonstrating that the method can be used to distinguish between lesions, and may also be helpful for downstream treatment [51].

## 4. Materials and Methods

MRI studies of CRL-2314 breast cancer cell lines and normal HTB-125 cells were performed. The MRI methodology was developed using a clinical scanner with a 1.5 T field. An attempt was made to use clinical MRI to track response to therapy in in vitro studies of breast cancer cell lines. Non-destructive cellular studies have been performed using the MRI method, and there is a chance to transfer the studies to in vivo studies in the future. As more and more cases of breast cancer have been seen in recent years, research may contribute to a better understanding of the drug and optimization of therapy.

The CRL-2314 line and culture media are from American Type Culture Collection (ATCC), PO BOX 1549, MANASSAS, VA 20108 USA, and were purchased under LGC standards (Lomianki, Poland). The sodium bicarbonate was from Honeywell Fluka; the freeze-dried bovine collagen-type fibrous powder from tendon was from Advanced BioMatrix (USA), and penicillin–streptomycin–neomycin stabilized, fetal bovine serum (FBS), and epidermal growth factor (EGF) were from Sigma-Aldrich and used to prepare the complete cell growth medium under sterile conditions in an Alpina laminar chamber. Both cell lines were cultured under standard conditions: 37 °C, 5% CO_2_ and 95% humidity.

The culture medium consisted of ATCC Hybri-Care Medium, catalog number 46-X (lgsstandards). To obtain a complete growth medium, the basal medium was supplemented with 30 ng/mL of mouse EGF (growth factor) and fetal bovine serum (FBS) from Sigma-Aldrich, (Sigma-Aldrich, USA) to a final concentration of 10%. Each cell culture was passaged on day 3 using Accutase Cell Detachment Solution (Corning, NY, USA) into five 70 mL tissue culture bottles (ThermoFisher Scientific, Waltham, MA, USA).

Custom-made glass bioreactors of our own design were used to prepare 3D cell cultures. “Fiber” was made from a blood collection capillary with a diameter of 2.30 mm from Saestest AG & Co., Nümbrecht, Germany, in which holes were made so that there was a possibility of accessing the culture medium. The capillary was then gas-sterilized and placed in a sterile bioreactor.

The bioreactor design includes an intracapillary (IC) space, which is the interior of the capillary, while the extracapillary (EC) space is where the cells inoculate in the bioreactor onto fibers and expand there. The cell culture medium during culture was in both the IC and EC spaces, intended to supply oxygen and nutrients to the). At the top of the bioreactor is a chimney in which a 0.22 μm filter was placed, so as to p cells revent external access by unwanted agents. The fiber was coated with 10 mL of collagen solution (1 mg collagen per 1 mL of phosphate-buffered saline (PBS) and 10 mL of fibronectin solution (10% in culture media). Each bioreactor was inoculated with 0.5 × 10^7^ cells through the rubber septum on the right-side port. After inoculation, the bioreactors were perfused using a peristaltic pump and maintained in a 5% CO_2_/95% air incubator. The perfusion of the bioreactor was delayed by 4 to 8 h to facilitate the adhesion of the cells to the fibers. After that time, the flow was started at the rate of approximately 5 mL/min and was increased over the next days to a rate of 14 mL/min.

## Figures and Tables

**Table 1:** Results of T_1_ and T_2_ relaxation times of two cell lines, CRL-2314 and HTB125, and samples of these lines with the addition of Trastuzumab (1) solution.

Cells	Treatment	Relaxation Times		Relaxation Times after 24 h	
		T_1_ (ms)	T_2_ (ms)	T_1_ (ms)	T_2_ (ms)
HTB-125	none	2914.96 ± 145.75	143.26 ± 7.16	2691.10 ± 134.56	152.95 ± 7.65
HTB-125	**1**	2885.11 ± 144.26	160.30 ± 8.02	2495.61 ± 124.78	153.29 ± 7.66
CRL-2314	none	2892.57 ± 144.63	154.10 ± 7.71	1801.66 ± 90.08	148.80 ± 7.44
CRL-2314	**1**	2509.04 ± 125.45	154.01 ± 7.70	1759.88 ± 87.99	143.35 ± 7.17

**Table 2:** Results of MR relaxation time measurements.

Cells	Treatment	T_1_ (ms)	T_2_ (ms)
HTB-125	none	2614.88 ± 130.74	126.27 ± 6.31
HTB-125	**5**	2117.76 ± 105.89	79.54 ± 3.98
CRL-2314	none	2603.95 ± 130.20	112.35 ± 5.62
CRL-2314	3	2565.18 ± 128.26	32.81 ± 1.64
CRL-2314	4	2505.52 ± 125.28	109.37 ± 5.47
CRL-2314	5	2382.18 ± 119.11	66.61 ± 3.33
cells CRL-2314	none	2266.9 ± 113.35	113.35 ± 5.67
cells CRL-2314	4	2157.53 ± 107.88	134.22 ± 6.71
cells CRL-2314	5	2137.64 ± 106.88	142.18 ± 7.11

## Data Availability

Data are contained within article.
